# Targeting type H vessels in bone‐related diseases

**DOI:** 10.1111/jcmm.18123

**Published:** 2024-02-14

**Authors:** Juan Xu, Shuang‐jian He, Ting‐ting Xia, Yu Shan, Liang Wang

**Affiliations:** ^1^ Outpatient Department Children's Hospital of Soochow University Suzhou China; ^2^ Department of Orthopaedics Suzhou Hospital, Affiliated Hospital of Medical School, Nanjing University Suzhou China; ^3^ Clinical Research Institute Suzhou Hospital, Affiliated Hospital of Medical School, Nanjing University Suzhou China; ^4^ Department of Orthopeadics Suzhou Ninth Hospital Affiliated to Soochow University Suzhou China; ^5^ Department of Orthopeadics The Fourth Affiliated Hospital of Soochow University Suzhou China

**Keywords:** angiogenesis, osteoarthritis, osteogenesis, osteoporosis, type H vessels

## Abstract

Blood vessels are essential for bone development and metabolism. Type H vessels in bone, named after their high expression of CD31 and Endomucin (Emcn), have recently been reported to locate mainly in the metaphysis, exhibit different molecular properties and couple osteogenesis and angiogenesis. A strong correlation between type H vessels and bone metabolism is now well‐recognized. The crosstalk between type H vessels and osteoprogenitor cells is also involved in bone metabolism‐related diseases such as osteoporosis, osteoarthritis, fracture healing and bone defects. Targeting the type H vessel formation may become a new approach for managing a variety of bone diseases. This review highlighted the roles of type H vessels in bone‐related diseases and summarized the research attempts to develop targeted intervention, which will help us gain a better understanding of their potential value in clinical application.

## 1 INTRODUCTION

Bone is a highly vascularized organ. The blood supply provides not only energy for skeletal muscle‐guided bone movements but also essential nutrients and trace elements for the survival and metabolic activities of various bone cells, which is pivotal for bone development, homeostasis and regenerative repair.^1^, ^2^, ^3^, ^4^, ^5^ Signalling crosstalk between the endothelial cells and surrounding bone cells creates a specific chemical microenvironment for the functional activities of multiple cells in bone.^6^ When vascular function is impaired or disrupted, multiple processes of bone metabolism can be affected.^7^, ^8^ Although the relationship between intraosseous angiogenesis and osteogenesis has long been recognized, the precise mechanism has been poorly understood previously. It is not until recently that the discovery of type H vessels, a particular subtype of intraosseous blood vessels double positive for CD31 and Endomucin (CD31^hi^ Emcn^hi^), shed a new light on this field.^9^ As the functions and related mechanisms of type H vessels continue to be revealed, our understanding of the relationship between intraosseous angiogenesis and osteogenesis is rapidly updating. At the same time, type H vessels are attracting increasing attention, especially in the aetiology and intervention of bone‐related diseases. Here, we reviewed the basic characteristics of intraosseous type H vessels and their essential roles in bone‐related diseases, with a view to further summarize their importance and potential clinical applications.

## 2 TYPE H VESSELS MAINLY LOCALIZE IN BONE

Type H vessels were identified by immunofluorescence staining when researchers tried to categorize multiple populations of endothelial cell (EC) with varied morphological and molecular properties in murine bone. They were mostly found in the metaphysis and endosteum area, and were generally straight, columnar and connected to the distal vessel loops or arches. Blood vessels in the diaphyseal area, on the other hand, showed weak positive staining for CD31 and slightly lower Emcn expression (CD31^lo^ Emcn^lo^, type L vessel). More notably, there were large numbers of Osterix, type‐I collagen and Runx2‐positive bone progenitor cells around type H vessels but almost none around type L vessels, suggesting the importance of type H vessels in osteogenesis.^9^, ^10^ Later study also confirmed the presence of type H vessels in human bone.^11^ In addition, investigations in mouse and human bone samples revealed that the number of type H vessels decreased significantly with increasing age.^9^, ^12^ Type H vessels identified in mandibular condyle also show age‐related decrease and are correlated with Osterix^+^ osteoprogenitor cells in condyle induced by mandibular advancement.^13^

The age‐dependent change of type H vessels in bone indicates that they are actively involved in skeletal development, which are supported by several studies of paediatric osteoporosis. Antenatal corticosteroid therapy suppresses Ezh2 expression by osteoclast precursors, resulting in reduced pre‐osteoclast number and PDGF‐BB secretion, which further leads to inhibited type H vessel formation and low bone mineralization.^14^, ^15^ Moreover, glucocorticoid treatments for chronic inflammatory and autoimmune conditions in children also have adverse effects on skeletal growth by inducing the senescence of type H endothelial cells.^16^ Other studies also identified Cathepsin K (Ctsk) inhibition as a potential preventive strategy against glucocorticoid‐induced osteoporosis in children for preservation of type H vessels and osteoblast.^17^ Other than corticosteroid‐induced osteoporosis, prenatal caffeine exposure also can lead to long bone dysplasia as a result of reduced type H vessels caused by the decreased expression of connective tissue growth factor by miR‐375.^18^

## 3 TYPE H VESSELS COUPLE ANGIOGENESIS AND OSTEOGENESIS

**FIGURE 1:**
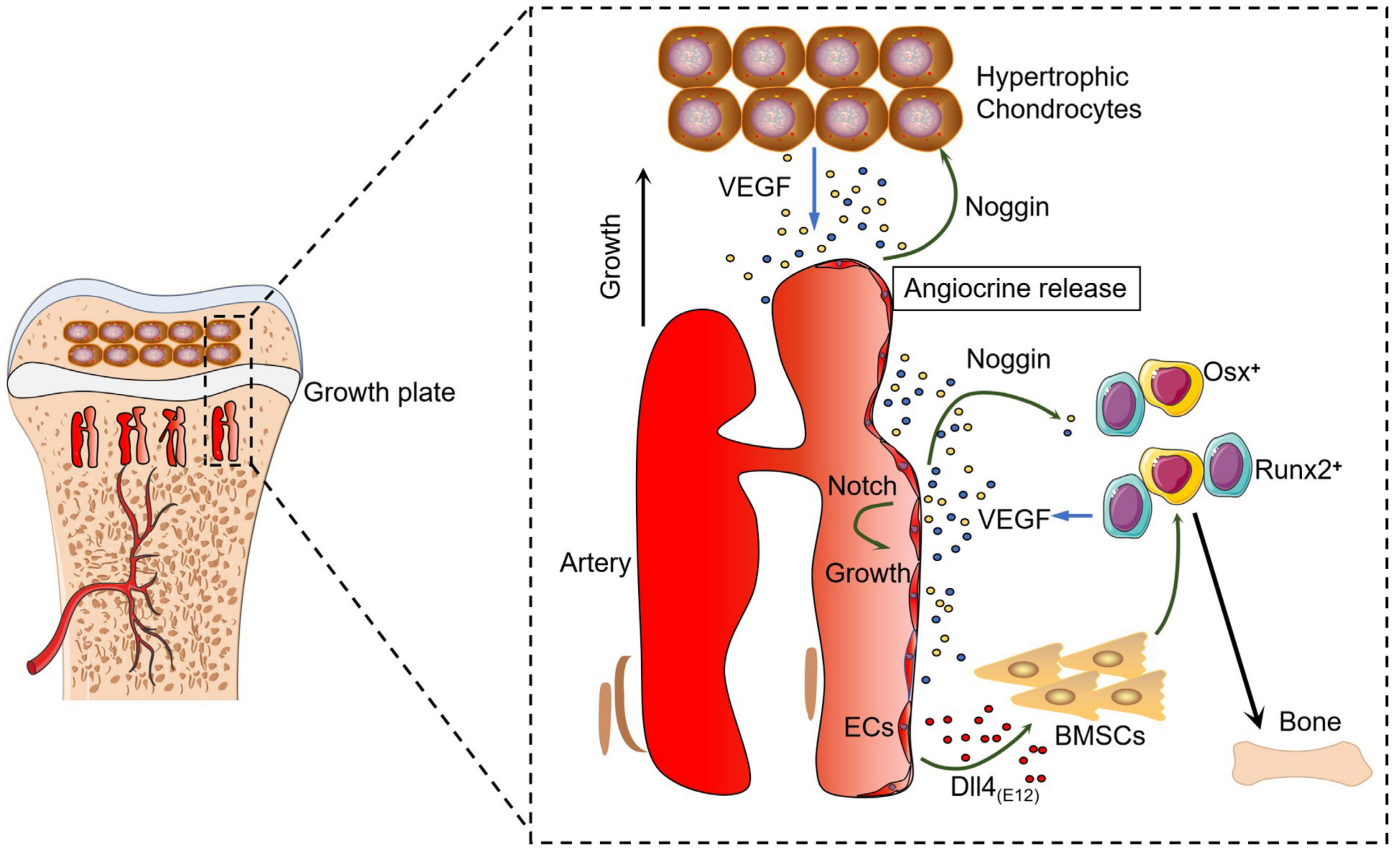
Type H vessels couple angiogenesis and osteogenesis and related molecular mechanisms. Type H vessels are abundant in the metaphasis next to growth plate chondrocytes. Large numbers of Osterix and Runx2‐positive bone progenitor cells are distributed around type H columnar vessels. Type H vessels expend towards growth plate chondrocytes. Type H endothelial cells secret Noggin that promotes Osterix^+^ and Runx2^+^ osteoprogenitors differentiation. Noggin can accelerate hypertrophy and maturation of chondrocytes, which in turn secrete VEGF to promote type H vessels formation. Notch ligand Delta‐like 4 [Dll4_(E12)_] also promotes the differentiation of mesenchymal stem cells into osteoblasts to increase bone formation. Bone vessels formation may be regulated by Notch signalling. Adapted from Saravana K. Ramasamy, Anjali P. Kusumbe, Lin Wang, et al. Endothelial Notch activity promotes angiogenesis and osteogenesis in bone. Nature, 2014, 507: 376–380.^19^

Along with the discovery of type H vessels, researchers have also identified their essential roles in coupling intraosseous angiogenesis and osteogenesis, a process that is critical for maintaining the homeostasis of bone metabolism.^9^ The coupling is manifested by the close localization of osteoprogenitor cells positive for Osterix and other markers. Vascular endothelial cells participate in the coupling process through a complex signalling crosstalk involving Notch, vascular endothelial growth factor (VEGF) and hypoxia‐induced factor 1‐alpha (HIF‐1α). Disruption of Notch signalling results in impaired type H vessel growth as well as skeletal defects due to decreased osteogenesis. Skeletal defects in Notch‐disrupted mouse models can be rescued by administration of recombinant Noggin, a key molecule secreted by EC and positively regulated by Notch signalling.^19^ Besides, bone formation in male mice can be also induced by a bone‐targeting, high affinity version of Notch ligand Delta‐like 4 [Dll4_(E12)_]. When used in combination with parathyroid hormone, Dll4_(E12)_ also enhanced trabecular bone formation in female and ovariectomized mice^20^ (Figure 1). HIF‐1α is highly expressed in type H ECs in young mice and gradually decreases with age, which is associated with age‐dependent bone loss. It involves in the coupling process to modulate intraosseous angiogenesis by controlling VEGF expression. EC‐specific knockdown of HIF‐1α induces a significant decrease of the metaphyseal and endosteal type H vessels.^9^ A further study also suggested that Yap1/Taz may be the downstream player of HIF‐1α to control angiogenesis in bone.^21^

On the other hand, bone cells can also secret a variety of molecules to participate in the coupling process, which makes the signalling crosstalk involved in the coupling of angiogenesis and osteogenesis more complicated. PDGF‐BB, secreted either by preosteoblasts or macrophage‐lineage TRAP^+^ cells, is reported to be pivotal for the coupling process, by inducing angiogenesis or recruiting periosteum‐derived cells.^22^, ^23^ Besides, overexpression of human PDGF‐BB in mouse endothelial cells also boosted both angiogenesis and osteogenesis in postnatal bones.^24^ Osteoblast‐derived Slit guidance ligand 3 (SLIT3), regulated by adaptor protein Schnurri3 (SHN3), is a proangiogenic factor of type H vessels involved in coupling of osteogenesis and angiogenesis as illustrated by genetic mouse models.^25^ CXCL9 secreted by osteoblast can interact with VEGF and prevent its binding to EC and osteoblast, thus abrogating Type H vessel formation and osteogenesis both in mouse bone and in vitro.^26^

## 4 TARGETING TYPE H VESSELS IN BONE‐RELATED DISEASES

The discovery of type H vessels as an active player in coupling intraosseous angiogenesis and osteogenesis has greatly broadened our understanding of bone modelling and remodelling. Meanwhile, as their roles in bone physiology and pathology are continuously being discovered, targeting type H vessels are becoming a new choice to intervene bone‐related diseases.

### 4.1 Osteoporosis

Osteoporosis, characterized by low bone mass, microstructural destruction and increased fragility and fracture risk, is the most common bone disease in human. Recently, studies have described type H vessels as sensitive markers for bone mass in human,^12^, ^27^ and more studies are investigating the effects of different treatments of osteoporosis on type H vessels.

**FIGURE 2:**
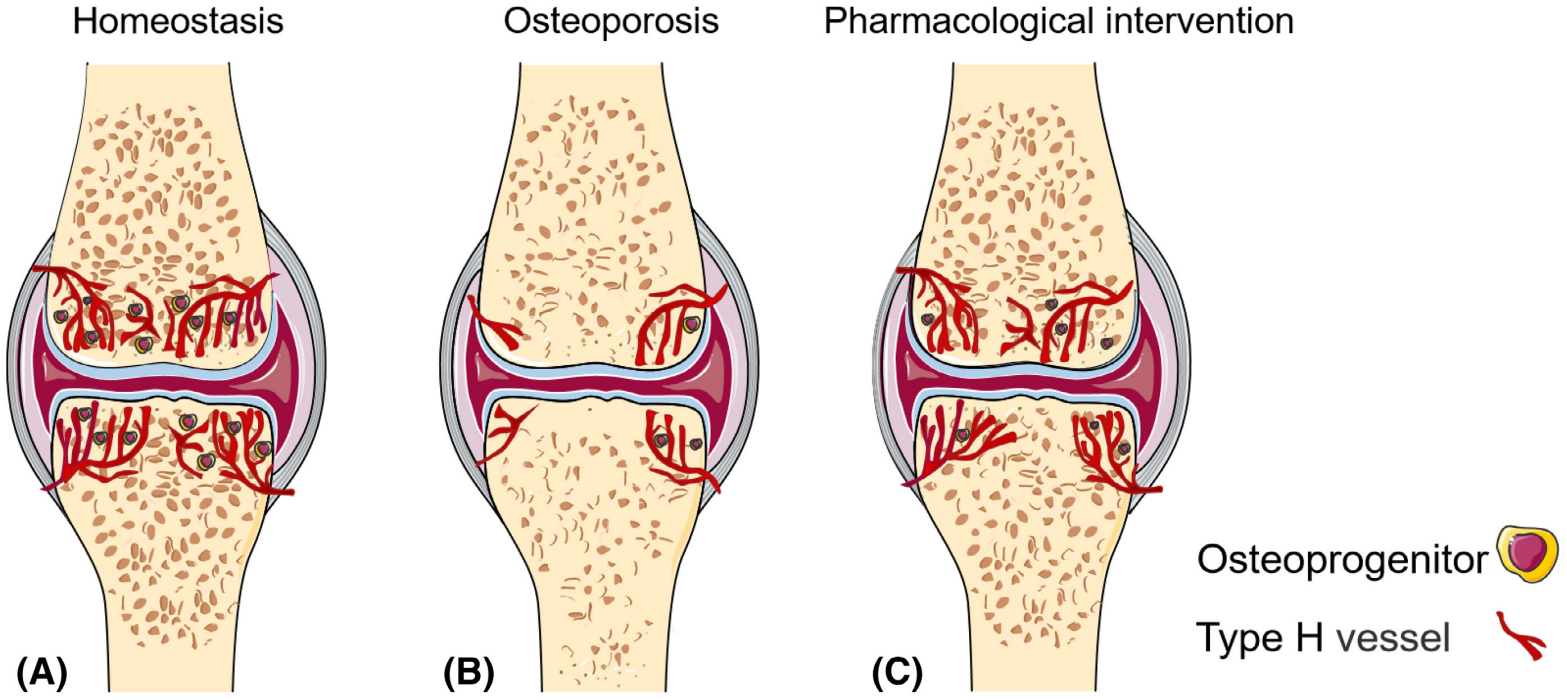
Type H vessels decrease in osteoporosis. In normal physiological homeostasis, an abundance of type H vessels is found in the metaphasis near the growth plate, which maintains perivascular osteoprogenitors (A). In the case of osteoporosis, bone mineral density decreases and microstructure is disrupted (B). Type H vessels and associated osteoprogenitors are strongly reduced in bone. Therapeutic intervention enhances the type H vessels formation, perivascular osteoprogenitors survival and then increases angiogenesis that promotes bone repair and regeneration (C).

Postmenopausal osteoporosis, also referred to as type I osteoporosis, is a type of primary osteoporosis and a common clinic disease, particularly in elderly women. Ovariectomized (OVX) mice and rats generated by surgical excision of both ovaries are generally used as animal models for simulating postmenopausal osteoporosis to study aetiology and evaluate medical interventions. Harmine, a natural tricyclic β‐carboline alkaloid, can prevent bone loss in OVX mice by promoting type H vessel formation, primarily by increasing the secretion of platelet‐derived growth factor‐BB (PDGF‐BB) by preosteoclasts.^28^, ^29^ Gushukang, a traditional herbal compound used in Chinese medicine for the treatment of osteoporosis, could also induce type H vessel formation via elevated HIF‐1α expression in mice.^30^ It was also discovered that intraperitoneally administered deferoxamine (DFO, an HIF‐1α agonist) significantly increased the number of intraosseous type H vessels and surrounding osteoprogenitor cells, resulting in significantly improved BMD in the femur of OVX mice^9^, ^31^ (Figure 2).

Aside from postmenstrual osteoporosis, the role of type H vessels in other types of osteoporosis was also investigated. Reduced type H vessels were observed in hindlimb‐unloading mice with bone loss, which can be rescued by *Panax quinquefolium* saponin treatment by increasing the secretion of VEGF and Noggin.^32^ In glucocorticoid‐induced osteoporosis (GIO) mouse model, genetic loss of Cathepsin K (Ctsk^−/−^) or Ctsk inhibitor treatment could prevent the bone loss and maintain the number of type H vessels by increasing PDGF‐BB secretion to augment VEGF signalling.^17^ In addition, several micro‐RNAs were also reported to be involved in the functions of type H vessels. Endothelium‐specific activation of miR‐497~195 stimulates type H vessel and bone formation in aged mice, suggesting that it may play a role in age‐related osteoporosis.^33^ MiR‐136‐3p targets PTEN and modulates type H vessel formation, and its expression is significantly decreased in alcohol‐induced osteopenia.^34^ MiR‐29cb2, a bone‐specific miRNA that is elevated in the peripheral blood of osteoporosis patients, plays an important role in angiogenesis‐osteogenesis coupling by regulating HIF‐1α activity and type H vessel formation via its target HIF‐3α.^35^ These molecules and related signalling pathways are potential targets for the treatment of osteoporosis.

Besides pharmacological interventions, certain physiotherapy measures have also been reported to be beneficial for type H vessel and associated bone formation. In OVX mice, pulsed electromagnetic field (PEMF) promotes osteogenesis coupled with type H vessel formation, which is mediated by HIF‐1α signalling.^36^ Mechanical loading also promotes type H vessel formation in OVX mice by inhibiting miR‐214‐3p expression in exosomes.^37^

### 4.2 Osteoarthritis

**FIGURE 3:**
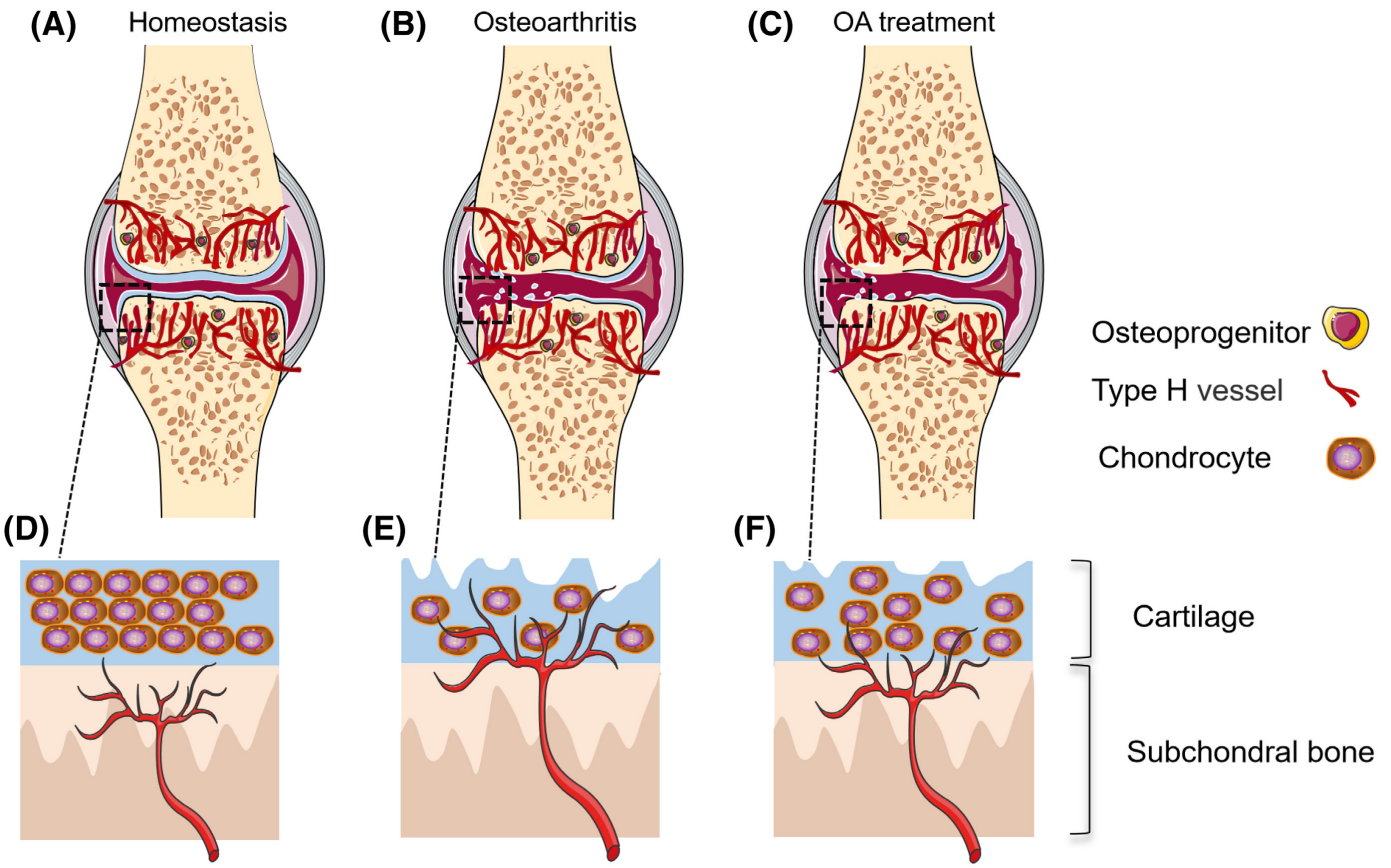
Increased subchondral bone vessels formation in osteoarthritis. Articular cartilage and angiogenesis are dynamically balanced in homeostasis (A, D). Osteoarthritis is characterized by degeneration of articular cartilage, structural damage of subchondral bone. Invasion of type H vessels deteriorates subchondral trabeculae and makes subchondral bone plate thin and irregular (B, E). Subchondral bone structure and type H vessels invasion into cartilage are improved during bone repair and regeneration (C, F).

Osteoarthritis (OA) is a chronic degenerative disease characterized by articular cartilage destruction, subchondral bone vascular invasion and aberrant bone remodelling.^38^, ^39^ It is now believed that neovascular invasion, represented by type H vessel formation, is crucial for the development of arthritis. In a mouse model of OA with meniscal instability, researchers discovered a significant increase of type H vessels in subchondral bone induced by increased VEGF secretion as a result of rapamycin complex 1 (mTORC1) signalling.^40^ Increased expression of osteopontin (OPN) in OA mouse model also promoted type H vessel formation in subchondral bone and accelerated the articular cartilage degeneration.^41^ The invasion of type H in subchondral bone was also confirmed in a mouse model of lumbar synovial arthritis by unilateral osteotomy of the lumbar synovial joint.^42^ With another rat OA model constructed by removing the anterior cruciate ligament, study showed that the level of phosphorylated polymerase in subchondral bone was dramatically elevated, which can be rescued by attenuating the formation of type H vessels with polymerase inhibitors.^43^ Halofuginone therapy can delay subchondral bone deterioration in a mouse OA model of anterior cruciate ligament (ACL) excision, with the main mechanism being suppression of the Smad2/3 TGF‐β signalling pathway, which attenuated subchondral neoangiogenesis of type H vessels.^44^ Defactinib treatment can also slow the progression of ACL excision‐induced OA by reducing the number of type H vessels and mesenchymal stem cells (MSCs) in subchondral bone.^45^ Therefore, type H vessel may play an essential role in the development of OA and inhibiting type H vessel formation in subchondral bone could be a new clinical strategy for OA treatment (Figure 3).

### 4.3 Fracture

Angiogenesis is undoubtedly crucial for the post‐fracture regenerative repair.^8^, ^46^, ^47^, ^48^ During fracture healing, vessels infuse osteoprogenitor cells into the fracture site, which further differentiate into osteoblasts and osteocytes, deposit in the bone matrix to form primitive bone tissues and finally become bone through mechanical shaping.^49^, ^50^ Timely and ordered vessel formation is required to support numerous activities in the regenerative repair of bone.^51^, ^52^, ^53^ A number of recent studies have discovered that various therapies can influence regenerative bone repair via altering the type H vessels, emphasizing the possibility of vascular intervention targeting type H vessels to promote fracture healing.

Osteoblast‐derived, but not exogenous EGFL6 enhances the osteogenic differentiation of bone marrow mesenchymal stem cells (BMSCs) and accelerates bone regeneration by stimulating type H vessel formation in a rat tibia distraction osteogenesis model.^54^ *Akkermansia muciniphila*, a gut probiotic, induces PDGF‐BB^+^ preosteoclasts and type H vessel formation in fracture healing by reducing intestinal permeability and alleviating inflammation in callus.^55^ Inhibition of stimulator of interferon genes (STING) enhances type H vessel formation and promotes bone healing after tibial fracture surgery.^56^ Aside from pharmacological agents, certain surgical grafts and physical therapies can also help with fracture repair by influencing the type H vessels. Low‐intensity pulsed ultrasound (LIPUS) has been shown to speed up fracture healing in a rat model of traumatic vertebral fracture by encouraging the regeneration of type H vessels on the surface of newborn trabeculae, bone marrow cavity and cartilage scab, as well as attracting large numbers of Osterix^+^ osteoprogenitor cells.^57^

### 4.4 Bone defect

Type H vessels are also critically involved in the regenerative repair of bone defects. In a study focussing on high bone mass (HBM) syndrome, a novel mutation in the long noncoding RNA Reg1cp was identified, which increased type H vessel formation by inhibiting Krüppel‐like factor 3 (KLF3) activity.^58^ Reversely, in a bone regeneration mouse model by surgical ablation of the trabecular bone, endothelial‐specific knockout of *Klf3* or treatment with ophiopogonin D (a *Klf3* inhibitor) promotes type H vessel neoangiogenesis and accelerates bone regeneration in the area of bone defects.^59^ Osteoblast‐specific and global EGFL6‐deficiency leads to compromised bone repair characterized by decreased type H vessels and osteoblast lineage cells in a tibial mono‐cortical bone defect model,^60^ which is consistent with the results obtained in fracture repair.^54^ Total flavonoids extracted from the Chinese herb *Rhizoma Drynariae* could also speed up the regenerative repair of bone defects in rats, with the main mechanism being the promotion of a large number of type H vessel formation in bone via PDGF‐BB and related pathways.^61^

Similar to fracture healing, some surgical grafts and physical therapies have also been found to accelerate the regenerative repair of bone defect by activating type H vessel formation. Surgical bone autograft made of poly‐GLP‐1 molecules can facilitate BMSC migration by transduction of Smad2 signalling pathway, leading to increased number of type H ECs in bones, which is helpful for bone regeneration.^62^ In a calvarial defect mouse model, silicified collagen scaffolds (SCSs) showed the ability to promote type H vessel formation and associated osteogenesis by immunomodulation of monocytes and cytokine release, resulting in enhanced recruitment of osteoprogenitor cells.^63^ Copper‐containing metal (CCM) promotes the generation of type H vessel during bone repair in drilling hole injures of mouse tibia by regulating the expression of PDGF‐BB from M2a macrophages.^64^ Filling bone defects with 3D‐printed irregular scaffolds containing recombinant human bone morphogenetic protein‐2 (rhBMP‐2) promotes bone repair in an aged rat model by promoting the formation of type H vessels and the recruitment of osteoprogenitor cells at the defective sites.^65^ Low‐level lase therapy (LLLT) is found to increase type H vessel formation in mBMSC/BCP grafts (bone marrow mesenchymal stem cells combined with biphasic calcium phosphate) implanted in mice, leading to enhanced bone tissue regeneration.^66^ Due to the active role of type H vessels in the bone regenerative repair, more and more tissue engineering efforts are being made to promote type H revascularization by targeting various signalling pathways (also summarized in Ref.^67^), which could be an important therapeutic strategy for accelerating bone regeneration.

### 4.5 Bone metastasis

Bone is a common site for metastasis of primary tumours such as those of the breast, lung and prostate.^68^ It is known that vasculature is critical for tumour metastasis to many organs, but the role of vasculature in bone metastasis is less understood, let alone type H vessels. When the disseminated tumour cells (DTCs) have infiltrated into bone marrow, their outgrowth is closely associated with the local microenvironment and they may remain in a dormant state as single cells or micrometastases.^69^ It was reported that a quiescent bone marrow niche could be characterized by the bone‐specific expansion of pericyte and also quiescence‐promoting secretome, which was associated with DTC dormancy. In response to radiation or chemotherapy, a bone‐specific expansion of pericytes was activated by type H vessel‐induced PDGF‐B signalling.^70^ In another mouse mammary tumour metastasis model, DTCs were found to preferentially localize adjacent to type H vessels in metaphyseal domain and metastatic lesion remodelled local vasculature to support tumour growth.^71^ The exact roles of type H vessel in DTC dormancy and reactivation still need to be further investigated.

## 5 CONCLUSION AND PROSPECTIVE

When the type H vessels were first identified, their correlation with aging was immediately noted,^9^ which was further verified in subsequent studies using clinical samples.^12^ Changes in type H vessels during aging also coincide with alterations in the bone marrow microenvironment (as reviewed in Ref.^72^). However, no detailed analyses have been done at the single‐cell level to illustrate the complex changes in intraosseous vessels during ageing. Recently, researchers have used innovative analytical tools to visualize the vascular changes during aging at single‐cell resolution in various tissues and also endocrine system. In addition to vascular density, artery and pericyte number quantifications, high‐throughput analyses including gene expression, cell–cell interactome and 3D spatial proteomic analyses were also performed to make a comprehensive characterization of the vascular changes in the ageing process.^73^, ^74^ If similar approaches can be applied to the analysis of the intraosseous vasculature, the age‐dependent changes in type H vessels in bone can be dissected at a single‐cell level to further promote our understanding of their significance in bone metabolism.

It is now obvious that type H vessels actively participate in bone modelling and remodelling by coupling intraosseous angiogenesis and osteogenesis, but their interactions with other players in intraosseous microenvironment still need to be further investigated. In this unique microenvironment, the level of oestrogen is especially essential for the health of both blood vessels and bones in female.^75^It was also reported that the lymphatic vessels in bone are also involved in the formation of type H vessels,^76^ which further connected the vascular network in bone and emphasized their importance in bone metabolism. The actual role of type H vessels may be more involved in the creation of a specific niche that orchestrates the local activities to support the homeostasis of bone metabolism.^72^, ^77^, ^78^, ^79^

The involvements of type H vessels in bone‐related diseases and regenerative repair make them a promising therapeutic target. The detailed mechanism underlying the shift of vascular status in bone remain incompletely elucidated, but quite a lot of therapeutic agents have already shown the potential to intervene bone metabolism by modulating type H vessel contents in bone. Although the type H vessel is not necessarily the direct effector, the active angiogenesis it reflects is critical in the management of bone disorders. In addition, the capacity of certain treatments to drive type H vessel alteration may become an essential indicator of their efficacy in the future.

## AUTHOR CONTRIBUTIONS

**Juan Xu:** Writing – original draft (lead). **Shuang‐jian He:** Writing – original draft (equal). **Ting‐ting Xia:** Visualization (lead). **Yu Shan:** Conceptualization (equal); writing – review and editing (equal). **Liang Wang:** Conceptualization (lead).

## CONFLICT OF INTEREST STATEMENT

The authors declare no competing interests.

## Data Availability

Data sharing is not applicable to this article as no new data were created or analyzed in this study.
